# Radial EUS Examination Can be Helpful in Predicting the Severity of Acute Biliary Pancreatitis

**DOI:** 10.1097/MD.0000000000002321

**Published:** 2016-01-22

**Authors:** Emrah Alper, Mahmut Arabul, Fatih Aslan, Cem Cekic, Mustafa Celik, Serkan Ipek, Belkis Unsal

**Affiliations:** From the Department of Gastroenterology, Izmir Katip Çelebi University, İzmir, Turkey.

## Abstract

We investigated the utility of noncontrast enhanced endosonography (EUS) in predicting the severity of acute pancreatitis (AP) during the first 72 to 96 h of admission.

In total, 187 patients with acute biliary pancreatitis were included. The patients were classified into 2 groups as having severe and mild AP according to the Modified Glasgow scoring and computerized tomography severity index (SI). The 158 cases with mild and 29 cases with severe AP had a similar age and sex distribution.

Although none of the cases with mild AP developed morbidity and death, of the cases with severe AP, 16 developed serious morbidities and 5 died. On EUS examination, we looked for parenchymal findings, peripancreatic inflammatory signs, free or loculated fluid collections, and abnormalities of the common bile duct and the pancreatic channel. Statistical analysis indicated a significant relationship between the severity of AP with diffuse parenchymal edema, periparenchymal plastering, and/or diffuse retroperitoneal free fluid accumulation, and peri-pancreatic edema. We also defined an EUSSI and found that the EUSSI had sensitivity of 89.7%, specificity of 84.2%, positive predictivity value (PPV) of 88.9%, negative predictivity value (NPV) of 91.2%, and an accuracy of 87.9% in the differentiation of mild and severe AP. We found that the EUSSI had an accuracy of 72.4%, sensitivity of 75.4%, specificity of 65.1%, PPV of 69.3%, and NPV of 73.1% for determining mortality.

Our data suggest that EUS allowed us to accurately predict the severity and mortality in nearly 90% of cases with AP.

## INTRODUCTION

The best management of patients with acute pancreatitis (AP) is mostly dependent on our ability to distinguish mild AP (MAP) from severe AP (SAP) during the early stages of this disease. For predicting the severity of AP, we use scoring systems such as Ranson, Imrie, and Apache II. The problem with these scoring systems is that they are burdensome, requiring multiple measurements that are often not available upon admission.^1,2^ A new simple scoring system, the BISAP score, provides a single point for 5 parameters, the prognostic accuracy of which has been reported to be similar to that of the other scoring systems mentioned above. However, most clinicians prefer to use computed tomography (CT) as a prognostic indicator as the extent of fluid collections or necrosis on CT has been correlated with the severity of the disease. However, contrast allergy, contrast-induced renal failure, and increased necrosis due to iodine have been reported to be shortcomings of this technique.^3^

Nonetheless, these scoring systems, including CT grading and the CT severity index (CTSI), have reached their maximal utility and novel models are needed to further improve predictive accuracy. Although endosonography (EUS) has limited use in the early evaluation of AP, we believe that a careful EUS examination can give important clues to diagnose complications of SAP. Contrary to what we know as its decreased accuracy in evaluating pancreatic parenchyma during an attack of AP, with the help of EUS we can obtain very important information on the pancreatic parenchyma, pancreatic duct, and peripancreatic regions in cases with severe pancreatitis. Herein, we retrospectively presented our data regarding EUS examination during AP and compared its significance with clinical and laboratory indices, CT grading, and CTSI in our cases.

## MATERIALS AND METHODS

We enrolled 187 patients with a diagnosis of acute biliary pancreatitis hospitalized and treated between the period of June 2011 and December 2014. The diagnosis of acute biliary pancreatitis was made within the setting of ongoing severe epigastric pain, hyperamylasemia (of >3 times the normal), elevated liver enzymes, hyperbilirubinemia (of >2 mg/dL) and stones in the gall bladder. In the laboratory we noted the hemogram, serum C-reactive protein (CRP), calcium, albumin, and urea and creatinine levels in all of our patients. Exclusion criteria included the presence of chronic pancreatitis, chronic liver disease, primary sclerosing cholangitis, malignancy in the pancreas and neighboring organs, anatomical obstacles to performing EUS examination, contrast allergy, pregnancy, and chronic kidney failure. Within 72 to 96 h after admission, pancreatobiliary EUS examination and CT were performed on each patient. We used modified Glasgow and Marshall scoring to classify the severity of AP and to assess organ dysfunction in the cases, respectively. We also added information obtained from the CT severity index (CTSI) to differentiate between the cases with MAP and SAP. The study began after obtaining approval from the local ethics committee. We received consent form from patients.

### EUS Examination

After 12 h of fasting, EUS examination was performed using a radial echoendoscopy device under sedation with intravenous 2 mg/kg propofol infusion with anesthesist. EUS was performed after positioning the patients on their left lateral sides. The second part of the duodenum was reached with the echoendoscope and the uncinate process was examined first. Subsequently, the echoendoscope was pulled back slowly and the head, body, and tail regions of the pancreas were evaluated. At each station, the pancreatic parenchyma, pancreas channel, choledochus, peripancreatic vascular structures, peripancreatic retroperitoneal region, and peripancreatic space neighboring the liver were inspected very carefully (Table 1).

**TABLE 1:**
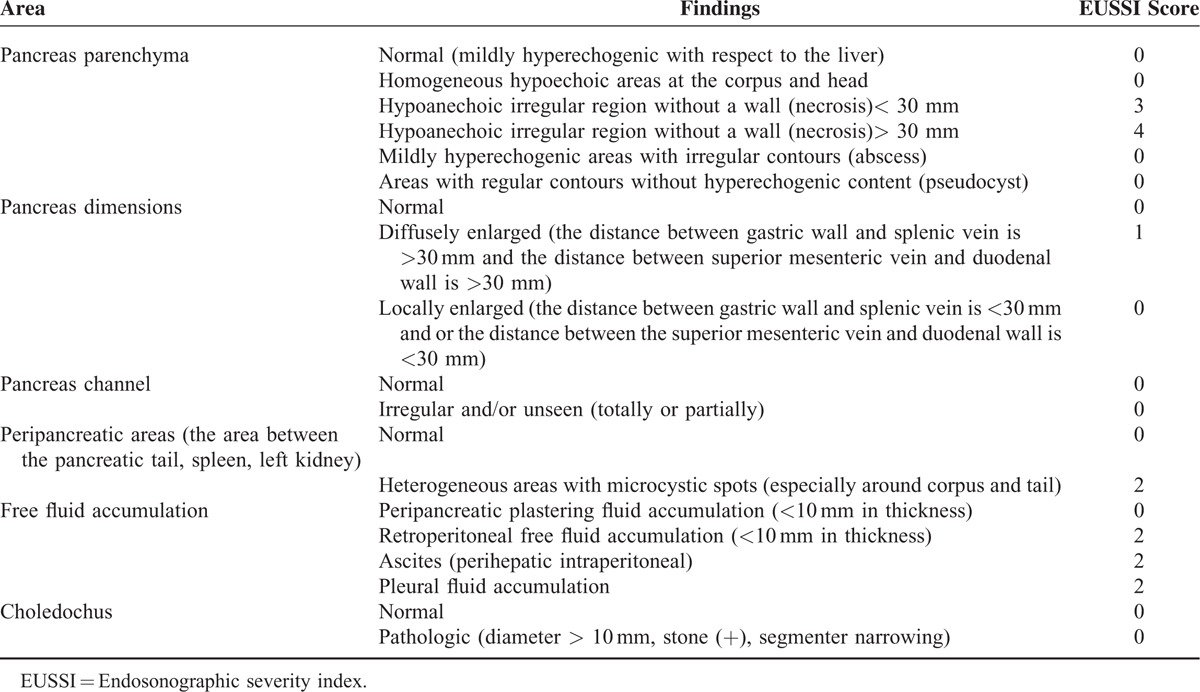
EUS Findings and Endoscopic Ultrasonography Severity Index (EUSSI) Scoring

### CT Imaging

CT with administration of oral and intravenous contrast was performed within 24 h after EUS examination. Dynamic imaging with CT was done during the arterial and venous phases according to the pancreatic protocol. Two radiologists highly experienced in abdominal tomography evaluated the CT images. On the CT images, the pancreas parenchyma and the existence of necrosis, inflammation extending to the peri-pancreatic fat tissue, and the presence of fluid collections were investigated. According to these findings, the CTSI was calculated.^4^ Dynamic CT was accepted as the gold standard examination technique in this study and we compared EUS and dynamic CT findings in our cases with SAP and MAP.

All of the subjects were classified into mild and severe AP groups according to the existing clinical and laboratory data with modified Glasgow scoring within the first 48 to 72 h of admission.^5^ We did a statistical comparison between these 2 groups with regard to demographic characteristics, laboratory values, hospitalization periods, and morbidity and mortality data. On the basis of EUS findings, although not validated, we created an EUS classification for severity of AP (Table 1), in which we adopted similar methodology to that of Balthazar et al.^6^ Accordingly, we scored the EUS findings on the basis of the logistic regression analysis results which are presented in Table 2 (if the *P* value was >0.05 the EUS parameter was scored as 0, if the *P* value was between 0.05 and 0.001 it was scored as 1, if the *P* value was <0.001 it was scored as 2, if the *P* value was <0.001 and if there was necrosis of <30 mm in size it was scored as 3, if the *P* value was <0.001 and there was necrosis of >30 mm in size it was scored as 4) and we calculated the EUS severity index (EUSSI) by summing up all of the scores in each case. We also compared the EUSSI with the clinical findings and the CTSI in all cases with AP. EUS scoring for necrosis was done similarly to the CT scoring for necrosis in the Balthazar classification.^6^

**TABLE 2:**
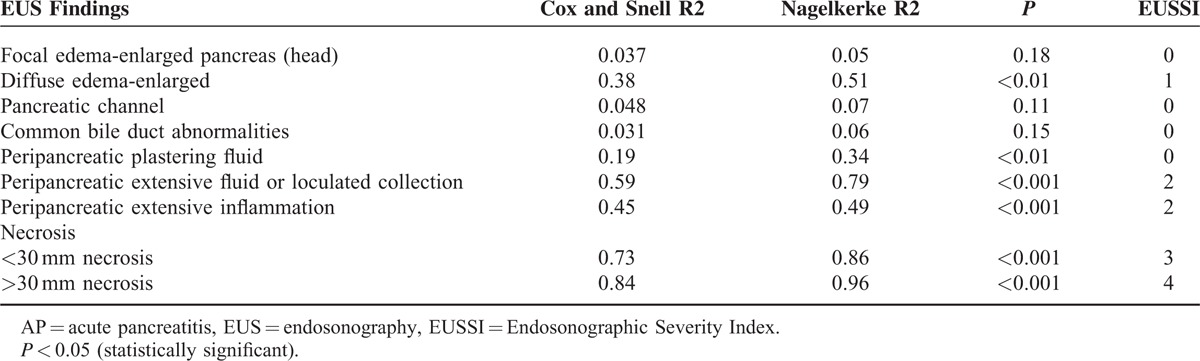
The Relation Between the Clinical Severity of AP and EUS Findings With EUSSI Scoring According to Logistic Regression Analysis

## STATISTICS

We used either the Mann–Whitney *U* test or *t* test for independent groups according to the dispersion pattern. For individual parameters, sensitivity, specificity, the negative predictivity value (NPV), the positive predictivity value (PPV), the positive likelihood ratio, and the diagnostic odds ratio (OR) were calculated for each imaging modality. Modalities were compared using McNemar's test of paired proportions. Correlations between CT, EUS, and laboratory parameters were evaluated with the Pearson correlation test. Multiple linear regression analysis was performed to evaluate the independence of the association between the EUS findings and SAP. The differences were considered to be statistically significant at *P* < 0.05.

## RESULTS

**FIGURE 1:**
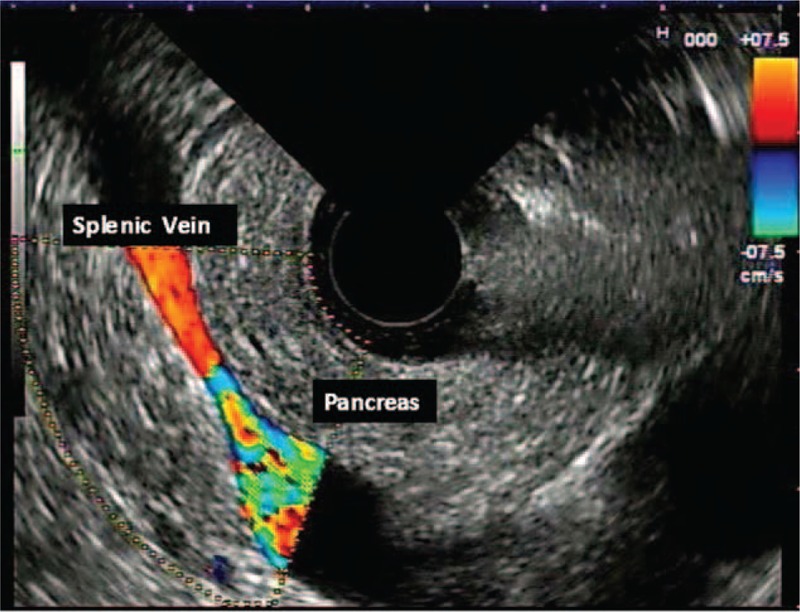
Normal pancreas.

**FIGURE 2:**
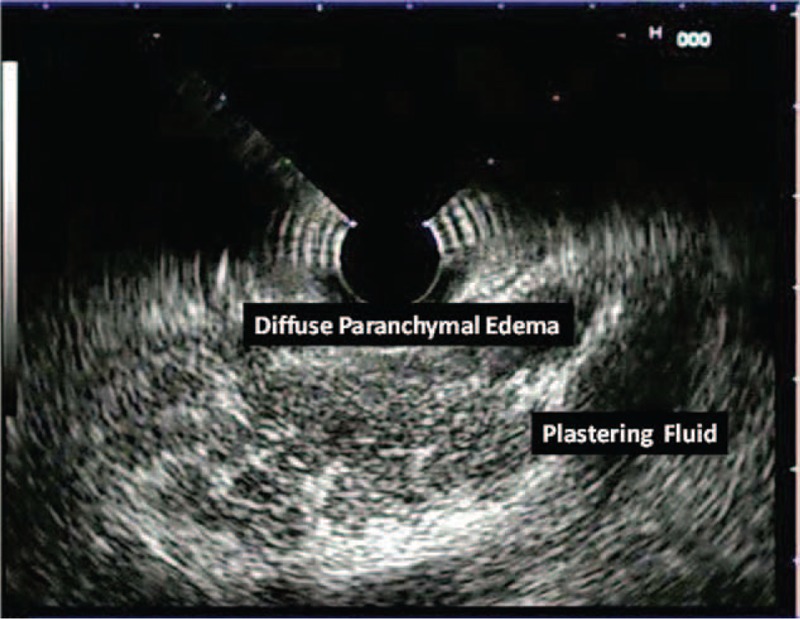
Diffuse parenchymal edema, diffuse enlargement, and plastering fluid collection are seen.

**FIGURE 3:**
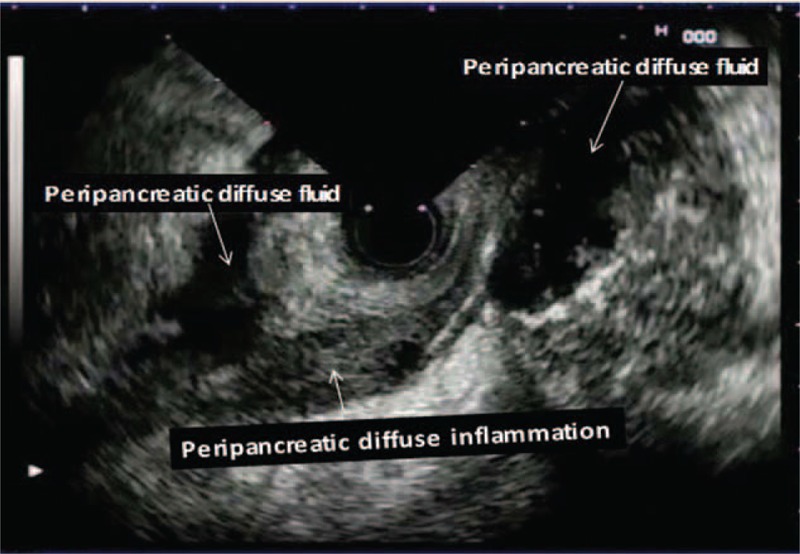
Peripancreatic localized excessive fluid and peripancreatic diffuse inflammation are noticed.

**FIGURE 4:**
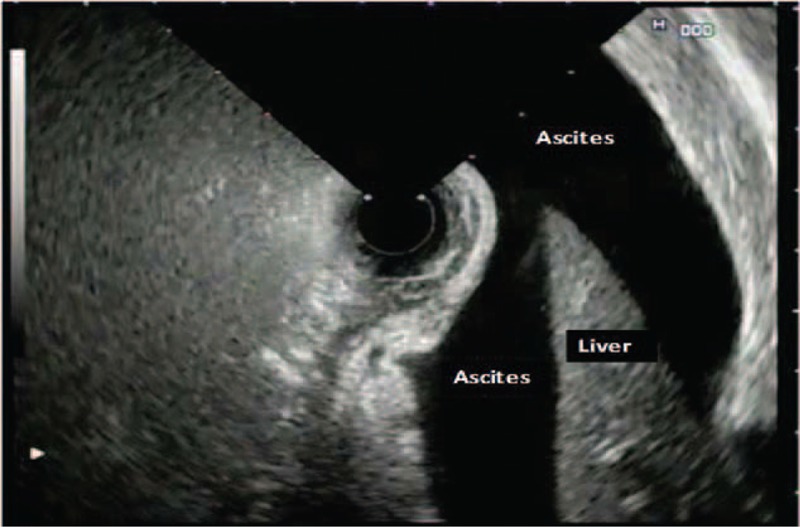
Pancreatic ascites.

**FIGURE 5:**
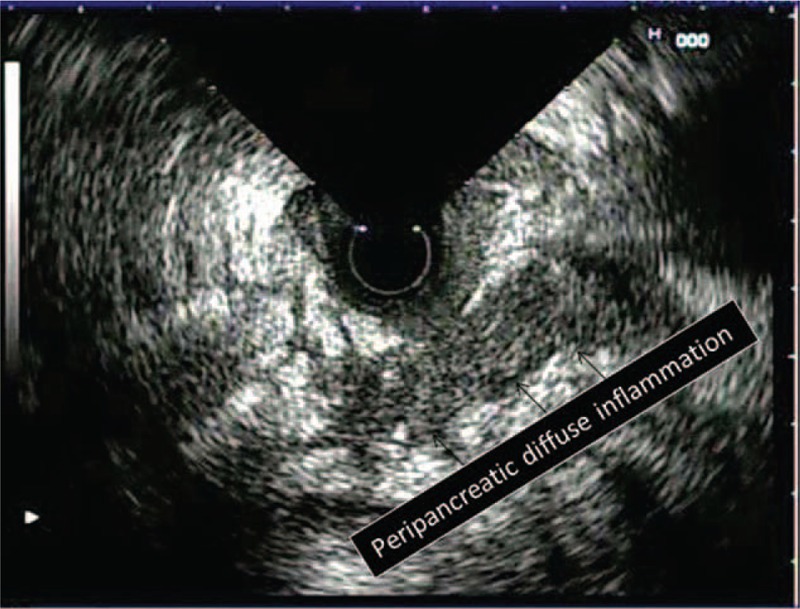
Peripancreatic diffuse inflammation is seen (arrowheads).

**FIGURE 6:**
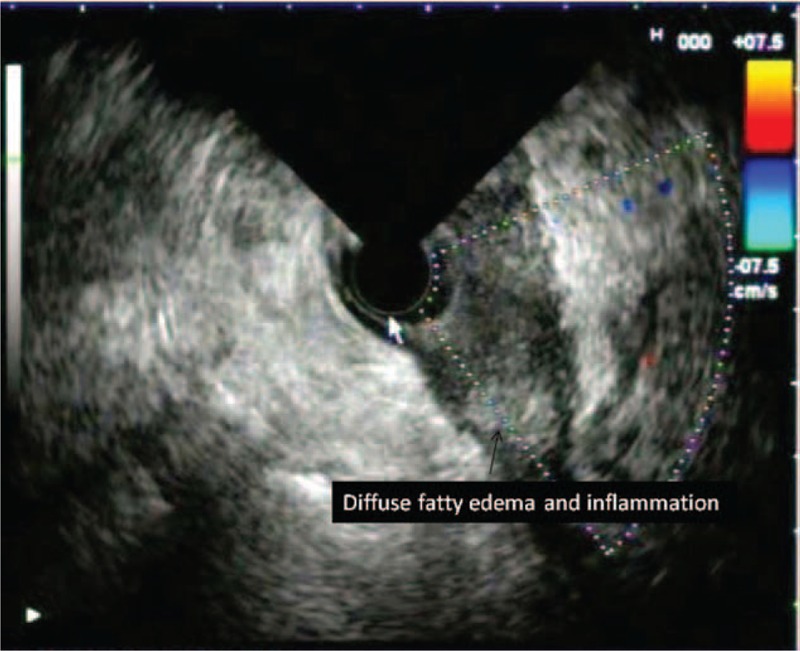
Diffuse fatty inflammation and fatty edema are demonstrated.

**FIGURE 7:**
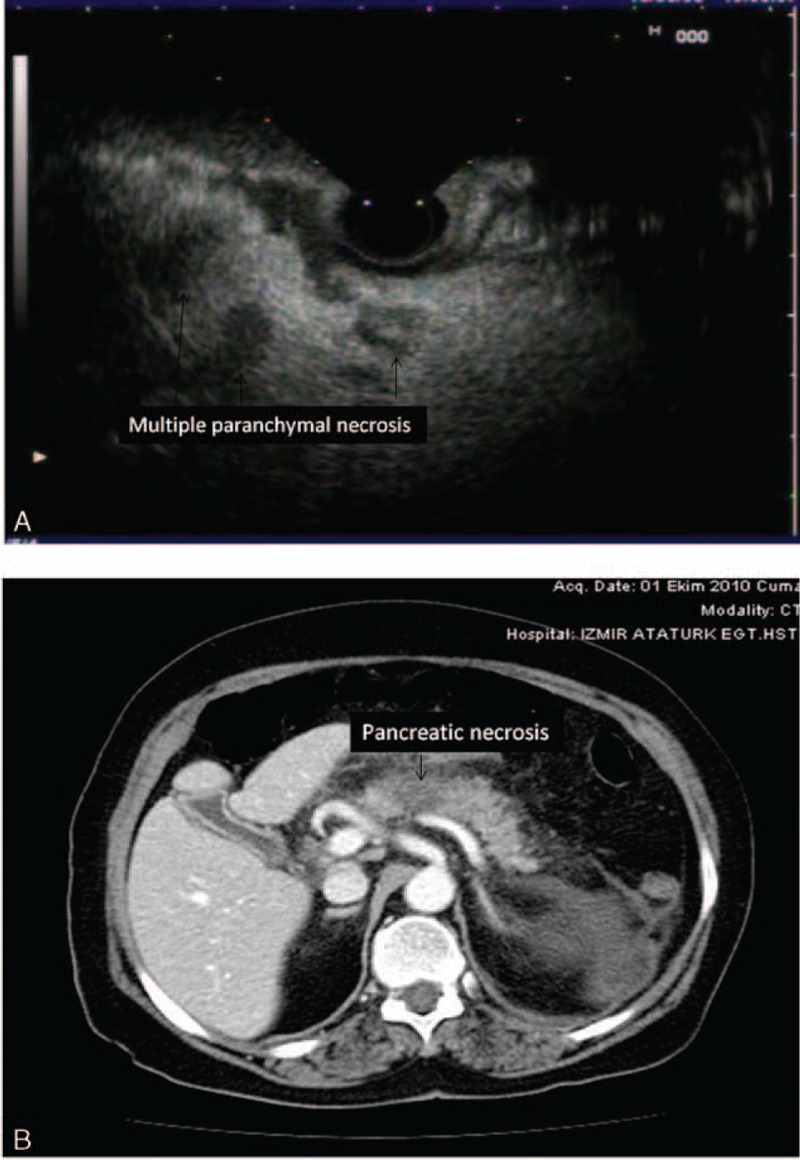
(A) Multiple pancreatic parenchymal necrosis (<20 mm) on EUS are seen (arrowhead). (B) CT view of the pancreatic necrosis (<20 mm) in the same patient. CT = computerized tomography, EUS = endosonography.

**FIGURE 8:**
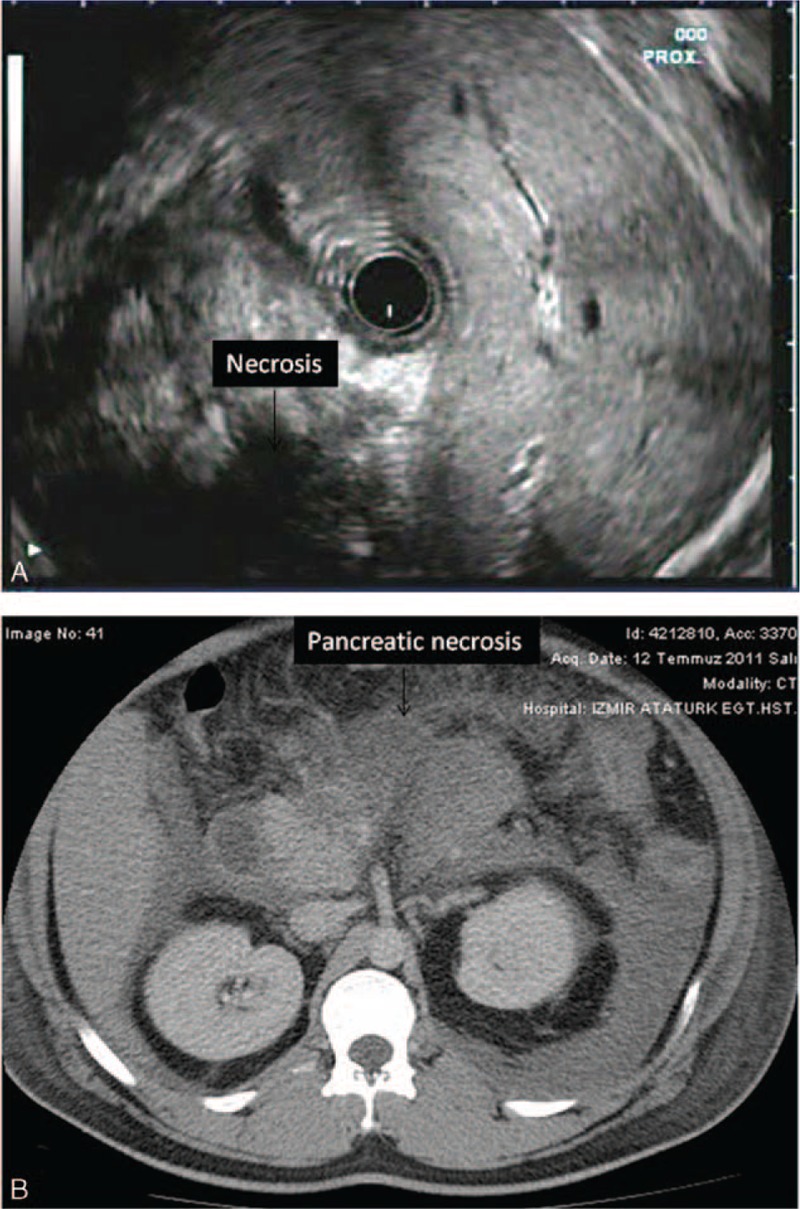
(A) Pancreatic necrosis (>30 mm) is shown. (B) CT appearance of the same patient indicating huge amount of parenchymal necrosis in the pancreas. CT = computerized tomography.

We enrolled 187 patients with a diagnosis of acute biliary pancreatitis. We classified our cases as SAP if the Balthazar CTSI indicated a value of >6 and/or modified Glasgow scoring showed >3 criteria. According to this classification, 29 patients (15.5%) had SAP, and 158 (84.5%) had MAP. There was no difference with regard to the age and sex distribution of the patients with mild and SAP. The mean CRP levels were significantly higher in the cases with SAP (34 ± 16 mg/dL) than in the cases with MAP (7 ± 6 mg/dL) (*P* < 0.05). Morever, the CRP levels correlated well with the EUSSI and CTSI (*r*:0.38, *P*:0.002). Twenty-five patients out of the 29 (85.2%) with SAP according to Glasgow scoring had a Balthazar CTSI ≥7. We used Marshall scoring to assess organ dysfunction in our cases at 48 to 72 h after admission to our clinics, which indicated a significant difference between the patients with MAP and SAP (0.5 ± 0.1 vs 3.1 ± 1.3, respectively, *P* < 0.05). Eight patients with SAP progressed to cardiac failure. Ten cases developed renal failure persisting for >48 h. Eleven patients with SAP developed several morbidities needing several surgical and or percutaneous procedures. Five of these patients died. No patient with MAP died or developed serious morbidities. Comparing patients with mild and SAP, there were also significant statistical differences regarding Marshall scores, morbidity and mortality figures (Figures 1–8), and the EUSSI and CTSl. Moreover, the mean hospitalization period was 17 ± 6 days (range: 49–12 days) in patients with SAP and 5 ± 1.8 days in patients with MAP (range: 4–8 days) (*P* < 0.001) (Table 3).

**TABLE 3:**
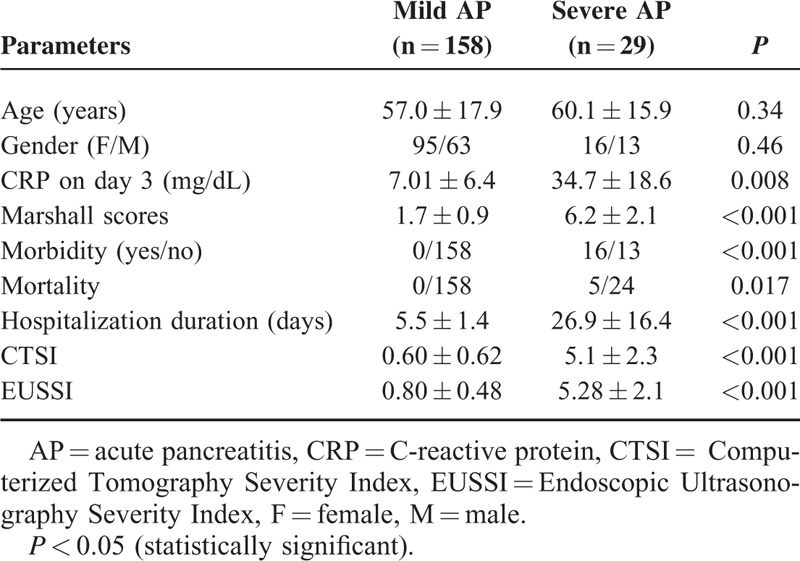
Comparative Analysis of the Patients With Mild and Severe AP

Twenty-one patients in the SAP group had pancreatic necrosis on dynamic CT imaging. In these patients, 9 of them had necrosis in >30% of the parenchyma and the others had necrosis ≤2 cm in size. In 18 of these cases, EUS demonstrated relatively hypo or anechoic appearances with irregular contours within the pancreas. In all of them, the extent and distribution of this EUS appearance in the pancreas was completely similar to the extent and distribution of the necrotic field demonstrated on dynamic CT imaging (Figures 1 and 2). However, 2 patients who had irregular pancreatic parenchyma with hypo-anechoic regions in the pancreas head and corpus did not have abnormal appearances on dynamic CT examination. When we accept dynamic CT as the gold standard for detecting pancreatic necrosis, the accuracy, sensitivity, specificity, and PPV and NPV of EUS examination for necrosis were highly striking as 92%, 85%, 94%, 79%, and 96%, respectively.

We compared the EUS findings in patients with MAP and SAP, which are presented in Table 4. According to the statistical analysis, there were significant differences between these 2 groups with regard to the number of patients having increased pancreatic dimensions, localized or diffuse edema in the pancreas, peripancreatic inflammation and fluid collections, ascites, pancreatic necrosis, disturbances in the main pancreatic duct structure, and common bile duct abnormalities (*P* < 0.05). Logistic regression analysis indicated a significant relationship between the severity of AP and some EUS findings such as diffuse parenchymal edema, periparenchymal plastering free-fluid collection, diffuse retroperitoneal free fluid accumulation, and peripancreatic edema in the nearby fat tissue (Table 4).

**TABLE 4:**
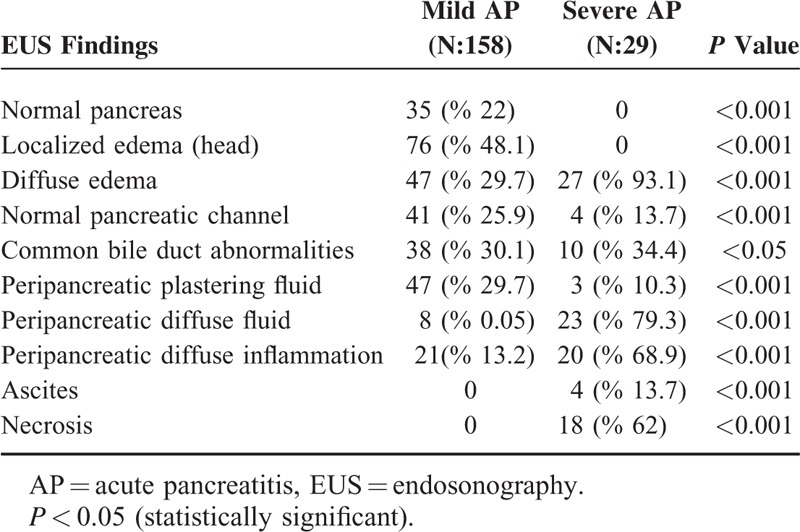
The EUS Findings in Cases With Mild and Severe AP

Dynamic CT was accepted as the gold standard examination technique in this study and we compared EUS and dynamic CT findings in our cases with SAP and MAP (Table 5). However, CT was not good at detecting abnormalities of the pancreatic channel and common bile duct and thus we did not compare the pancreatobiliary duct abnormalities seen on EUS and CT. Twenty-three of the 29 cases (79.3%) with SAP had EUS findings consistent with severe disease such as necrosis and/or free large or loculated peripancreatic fluid collections. Statistical analysis revealed that there was no difference in the ability to differentiate MAP from SAP with CT and EUS (Table 1).

**TABLE 5:**
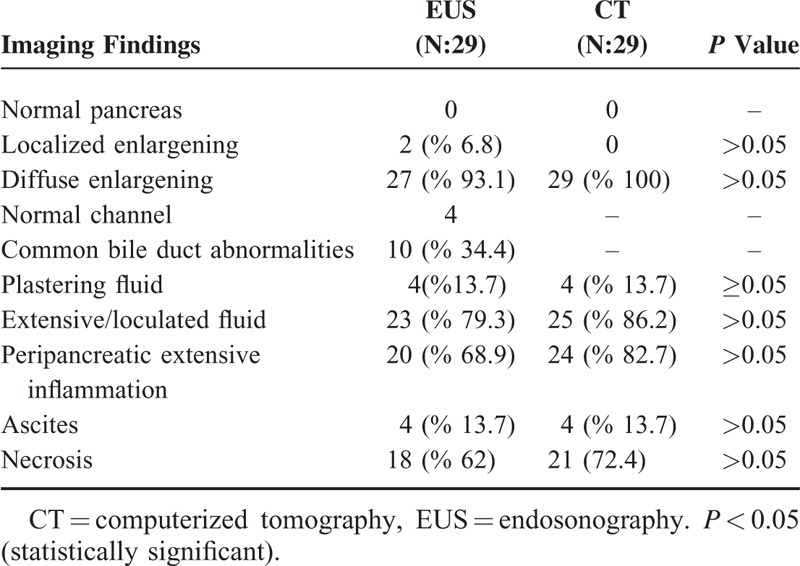
The Comparison of CT and EUS Imaging Findings

We evaluated extrahepatic bile ducts with EUS at all patients. There were 10 patients (34.4%) with common bile duct (CBD) stones in the SAP group at EUS investigation whereas were 38 patients (30.1%) in the MAP group. We detected CBD dilatation (>10 mm) without stone in total 21 patients (11%). Bile duct dilatation was considered to be due to the pressure effect of an inflamed and edematous pancreas on the distal CBD in these patients. There were no statistically significant differences in the biliary EUS findings between the patients with mild and SAP.

Nine cases with SAP had loculated larger or extensive retroperitoneal fluid accumulation and 4 of them had ascites, 8 had extensive peri-pancreatic inflammation, and 7 had diffuse edema and pancreatic enlargening. Eleven patients had extensive pancreatic parenchymal necrosis on dynamic CT examination. In these cases, EUS also showed hypoechoic areas in the pancreas of >30 mm in size which were distributed at the same localizations where the dynamic CT indicated necrosis.

We defined the EUSSI in cases with SAP according to the statistical results of the logistic regression analysis of the EUS findings noted in Table 2. Moreover, the presence of diffuse and/or large loculated peripancreatic fluid accumulation and diffuse peripancreatic inflammation on EUS was found to be highly accurate in differentiating between mild and SAP. The EUSSI had sensitivity of 89.7%, specificity of 84.2%, a PPV of 88.9%, an NPV of 91.2%, and accuracy of 87.9% in the differentiation of mild and SAP. A statistical analysis for mortality indicated that the CTSI had accuracy of 76.9%, sensitivity of 82.2%, specificity of 69.2%, a PPV of 71.7%, and an NPV of 78.6%. Likewise, the EUSSI had accuracy of 72.4%, sensitivity of 75.4%, specificity of 65.1%, a PPV of 69.3%, and an NPV of 73.1% for determining mortality (Table 6).

**TABLE 6:**
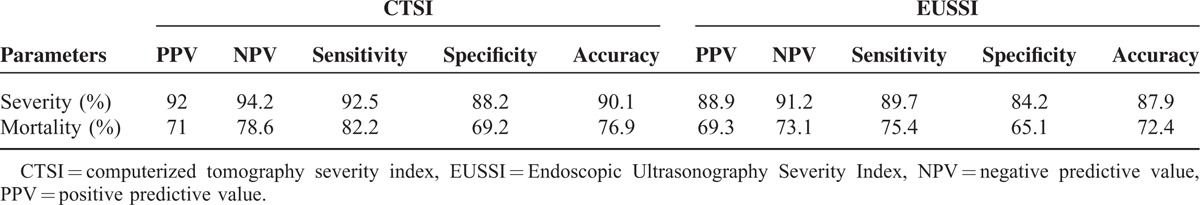
The CTSI and EUSSI Data About the Severity and Mortality of Acute Pancreatitis

## DISCUSSION

The radiologic severity of acute biliary pancreatitis is primarily determined using dynamic contrast enhanced CT examination. However, the contrast agents used during this examination have potential risks of nephrotoxicity and even exaggeration of the pancreatic disease in a substantial number of patients. There are few studies in the literature in which pancreatic parenchyma were evaluated with EUS during AP. Classically, EUS is primarily applied for suspicion of bile duct stones in patients presenting with AP. Another indication for EUS in such cases is to detect any mass lesion in the pancreas as a cause of AP. The most important limitation of noncontrast enhanced EUS in this clinical setting is known to be its inability to detect parenchymal necrosis. However, in 1 report investigating any other possible diagnostic role of noncontrast enhanced EUS in cases with AP.^7^ The authors described hypoechoic appearances in the pancreatic parenchyma as necrotic foci, which were confirmed by dynamic CT. In this report, EUS was proposed to have similar efficacy to that of dynamic CT in differentiating between edematous and necrotizing pancreatitis. Another report by the same authors described a high success rate in detecting the existence of pancreatic necrosis by EUS examination in cases with SAP.^8^ In our study, we detected intraparenchymal hypo and/or anechoic regions with irregular borders on EUS examination in cases with SAP. These regions were confirmed to be areas of necrosis by dynamic CT examination undertaken in accordance with pancreatic protocol revealing perfusion defects in these localizations. We classified such areas noticed by EUS examination to be more or less than 30 mm due to the inability of EUS to provide a view of all of the pancreas tissue at the same cross-sectional imaging. As a result, we found an overall efficacy of EUS in detecting necrosis of ∼92%. Another study concentrated on the ability of EUS to discern the severity of AP.^9^ Specifically, pancreatic inhomogeneity was reported to be a good predictor of SAP. In our study, we found that the peripancreatic retroperitoneal fluid collection localized around the pancreatic corpus and tail junction had an association with the severity of pancreatitis if its size was >10 mm (SAP 79.3% vs. MAP 0.05%, *P* < 0.05). Thus, the accuracy of EUS was found to be ∼79.3 % in predicting the severity of AP in our study.

Peripancreatic inflammation was easily detected on EUS and was seen as heterogeneous, well-demarcated, and mildly hypoechoic areas containing microcystic spots. We detected such appearances in 68.9 % of our cases with SAP and 25.8% of the cases with MAP. However, these appearances were more extensive and large in the SAP cases. Dynamic CT examination revealed that such areas were inflammed peripancreatic fatty tissue. Similarly, EUS findings reflecting peripancreatic inflammation were more common in cases with SAP than in the cases with MAP (*P* < 0.001).

EUS reveals diffusely enlarged and hypeochoic pancreas parenchyma in 75% of cases with edematous pancreatitis.^10^ An edematous and enlarged pancreas is seen as mildly hypoechoic and mildly heterogeneous on EUS examination. CT revealed diffuse parenchymal edema in all of the cases with SAP, whereas EUS showed diffuse edema in 93.1% of these cases. Only 10% of the cases with MAP had signs of diffuse edema and enlargening. Nevertheless, 9 cases with SAP with no signs of necrosis or loculated fluid collection did not develop any morbidity and mortality.

AP leads to pancreatic channel injury resulting in a rush of pancreatic enzymes into the peripancreatic region. This obviously contributes to the severity of pancreatic disease.^4,11^ Generally, CT cannot delineate pancreatic channel anatomy, whereas EUS obtains clearcut images of the whole anatomy of the pancreas channel throughout the pancreas. In our study, EUS showed a normal-appearing pancreas channel in a quarter of this cohort. Indeed, EUS could not detect the pancreas channel in 64% of the cases with mild pancreatitis and 86% of the severe cases. However, statistics revealed that the predictivity of this finding for SAP is relatively low (*P* < 0.05). We believe that this was because of edema in the pancreas. In the presence of necrosis, we could follow the pancreas channel up to its site of entry into the necrotic area. There were 6 such cases with large retroperitoneal free fluid accumulation. We obtained ascitic fluid samples via percutaneous from these patients and ascitic amylase levels were found to be >5000 IU/L in these cases. The ERCP examination revealed pancreatic leakage in all of these patients. Three of these patients died during the course of the disease. Due to the low number of patients, we did not perform any statistical analysis on pancreatic necrosis and a sudden cut in the pancreatic channel on the EUS images with regard to predictivity for the severity of AP. In our study, we also investigated the biliary tree as well as pancreatic parenchyma, the pancreatic channel, and the peripancreatic region by EUS. Other than existing choledocholithiasis in some of our patients we observed a sudden cut off in the distal bile duct due to pressure from the edematous pancreas tissue. However, we could not detect a significant correlation between bile duct abnormalities and the severity of pancreatitis.

One of the main purposes of the present study was to form an EUSSI similar to the CTSI proposed by Balthazar in 1990.^6^ The statistical data showed us that the sensitivity, specificity, PPV, NPV, and accuracy of the EUSSI were not significantly different than those of the CTSI in the differentiation of MAP and SAP. Both the CTSI and EUSSI correlated well with serum CRP levels. Similarly, the statistical data for the EUSSI in predicting mortality was as good as that of the CTSI. Considering the potential risks associated with contrast enhanced CT investigation such as radiation exposure and contrast-induced renal and pancreatic toxicity, EUS examination provides us with many pancreatic parenchymal and ductal data without such risks. Although contrast-enhanced EUS seemed to be more valuable in identifying necrotic areas in acute pancreatitis, ultrasonic contrast materials are expensive and are not available in many countries. Nevertheless, our data also suggest that even noncontrast enhanced EUS data can easily be used to form an EUSSI, which allows us to accurately predict the severity and mortality in nearly 90% of cases with AP during the first 72 to 96 h of admission.
